# Spatial distribution of cuticular drusen and its association with category-specific progression risk in intermediate AMD

**DOI:** 10.1038/s41433-026-04631-w

**Published:** 2026-06-19

**Authors:** Jianfeng Huang, Muneeswar Gupta Nittala, Giulia Corradetti, Yu-Chien Chung, Alberto Quarta, Rouzbeh Abbasgholizadeh, Ceren Soylu, Shinichiro Chujo, Swetha Bindu Velaga, Srinivas R. Sadda

**Affiliations:** 1 Doheny Eye Institute, Pasadena, CA USA; 2 Department of Ophthalmology David Geffen School of Medicine, University of California Los Angeles, Los Angeles, CA USA; 3 Department of Ophthalmology, Beijing Hospital, National Center for Gerontology, National Clinical Research Center for Gerontology, The Key Laboratory of Geriatrics of NHC, Institute of Geriatric Medicine, Chinese Academy of Medical Sciences, Beijing, PR China; 4 Department of Ophthalmology, Fu Jen Catholic University Hospital, Fu Jen Catholic University, New Taipei City, Taiwan; 5 Department of Ophthalmology, Mie University Graduate School of Medicine, Tsu City, Mie Japan

**Keywords:** Retinal diseases, Macular degeneration

## Abstract

**Objectives:**

To investigate the spatial distribution pattern of cuticular drusen using en face OCT and determine its relationship with 2-year progression of age-related macular degeneration (AMD).

**Methods:**

This study included 87 eyes from 57 participants with intermediate AMD and cuticular drusen enroled in the Amish Eye Study who completed two years of follow-up. Multimodal imaging, including volume spectral-domain OCT, was performed. Density of cuticular drusen was quantified on en face OCT across three Early Treatment Diabetic Retinopathy Study (ETDRS) grid zones using ImageJ. K-means clustering analysis was used to categorize distribution patterns. Firth’s penalized logistic regression evaluated association between cuticular drusen distribution categories and progression to late AMD at 2 years.

**Results:**

Three spatial phenotypes of cuticular drusen were identified: central-dominant (57.5%), outer-macular-dominant (32.2%), and diffuse (10.3%). Over two years, five eyes progressed to late AMD, four of which belonged to the outer-macular-dominant group. In unadjusted Firth regression, the outer-macular-dominant phenotype was associated with higher odds of progression than the other phenotypes (OR 7.16; 95% CI 1.24–74.20, *p* = 0.027). After adjustment for subretinal drusenoid deposits (SDDs) and large drusen, the association between spatial phenotype and progression remained (OR 11.53; 95% CI 1.62–169.00; *p* = 0.013), while SDDs were also associated with progression (OR 9.26; 95% CI 1.36–78.65; *p* = 0.023).

**Conclusions:**

Clustering of en face OCT–derived spatial features identified distinct cuticular drusen phenotypes within the macula. The outer-macular-dominant phenotype may be associated with an increased likelihood of AMD progression in this exploratory analysis.

## Introduction

Gass et al. [1] first described cuticular drusen as small, round, yellow nodules clustered in the macula and in the midperiphery of the fundus. On fluorescein angiography, the lesions were shown to have characteristic pattern described as a “stars-in-the-sky” appearance [2]. Similar to hard and soft drusen, cuticular drusen are located between the basal lamina of the retinal pigment epithelium (RPE) and the inner collagenous layer of Bruch’s membrane [3].

Cuticular drusen represent a phenotype of clinical relevance in the spectrum of AMD and may have specific genetic implications such as mutations in the CFH gene [4–8]. They are distinguished by their composition, morphology, and spatial distribution, and these characteristics were suggested to confer a unique risk for the development of GA and neovascularization in a previous study [9]. However, the impact of cuticular drusen on AMD progression remains controversial. Kai et al. indicated that individuals with the cuticular drusen phenotype had neither a higher nor lower risk of developing late AMD over 3 years and were not associated with a difference in rate of visual sensitivity decline compared with those without this phenotype [10]. Similarly, a recent study of our team found that the presence of cuticular drusen may not be the risk factor for developing atrophy within a 2-year period [11].

The reason for these apparent discrepant results regarding the impact of cuticular drusen on AMD progression may due to differential effects of different subtypes of cuticular drusen. Currently, two different classification systems have been used for categorizing cuticular drusen, one based on OCT imaging as described by Balaratnasingam et al. [9] and the other one based on colour fundus photographs (CFPs) as outlined by Sakurada et al. [4]. According to the study of Sakurada et al., phenotype 2 which manifested scattered cuticular drusen, extending into the periphery and phenotype 3 which was associated with large drusen ( > 200 mm) were reported to have a significantly higher risk of progression to geographic atrophy (GA) and macular neovascularization (MNV) than phenotype 1, which demonstrated a cluster of numerous small lesions, concentrated centrally and thinning out circumferentially [4].

Multimodal imaging including en face OCT provide new opportunities to more precisely and quantitively study the spatial distribution of drusenoid lesions and has provide novel insights into their pathophysiology [12, 13]. To our knowledge, previous studies have not yet used en face OCT to precisely analyse the spatial pattern of cuticular drusen. Our study was designed to investigate the spatial distribution of cuticular drusen using en face OCT and to study the relationship between different spatial patterns and the 2-year risk for progression to late AMD.

## Materials and methods

### Participants

All participants with intermediate AMD (based on Beckman criteria [14] applied to colour photographs) enroled in the Amish Eye Study, who also had a diagnosis of cuticular drusen, were included in this study. The Amish Eye Study is a National Eye Institute (NEI)supported clinical research investigation initiated to identify biomarkers of OCT for earlier stages of AMD in a family-based cohort, as well as to identify novel phenotypic and genetic biomarkers of AMD. Detailed descriptions of the overall Amish Eye Study including demographic and multimodal imaging features can be found in previous studies [15–17]. Written informed consent was obtained from all participants prior to enrolment. The study protocol was approved by the institutional review boards of the

University of California, Los Angeles; Case Western Reserve University; the University of Pennsylvania; and the University of Miami (IRB No. IRB-15-000083). All aspects of the study adhered to the tenets of the Declaration of Helsinki.

### Imaging protocols

Inclusion criteria consisted of eyes receiving multimodal imaging, including colour fundus photography, spectral-domain optical coherence tomography, consistent with established imaging protocols for AMD assessment [15, 16]: Heidelberg Spectralis HRA + OCT (Heidelberg Engineering, Heidelberg, Germany) consisted of a 512*97 macular volume cube (97 B-scans and 512 A-scans per B-scan, ART of nine frames) over a 6 mm * 6 mm field centred on the fovea; fundus autofluorescence (FAF) using Spectralis HRA, and near infrared reflectance using Spectralis HRA/OCT. Eyes were excluded if OCT demonstrated features of late AMD, if concomitant retinal pathology other than AMD was present, or if image quality was insufficient for analysis. Poor image quality was defined as significant media opacity or a signal strength of less than 20 dB on Spectralis OCT, consistent with the quality threshold used by the Doheny Image Reading Centre for acceptable images.

### Grading for cuticular drusen

**Fig. 1:**
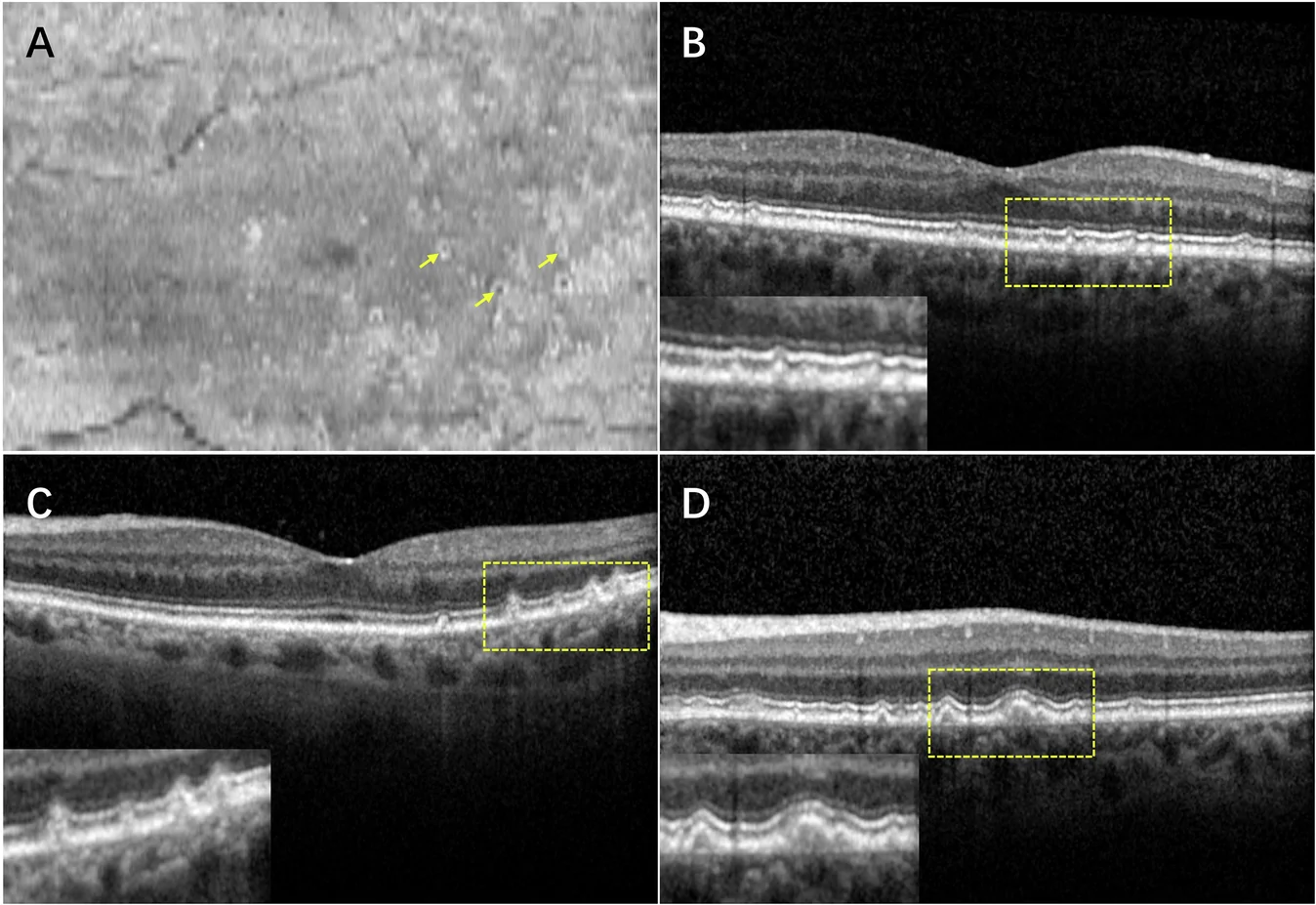
Representative images of CD on en face OCT and OCT B-scans. Highmagnification images of OCT findings are provided in the insets. **A** En face OCT; **B** Shallow elevations of the RPE–basal lamina complex; **C** The classic saw-tooth configuration with a hyporeflective internal core; **D** Broad, mound-shaped RPE–BL elevations. Arrows: Donut-like pattern of CD.

Traditionally, the identification of cuticular drusen has relied on clinical examination or colour fundus photography (CFP), with fluorescein angiography (FA) demonstrating the characteristic “stars-in-the-sky” pattern produced by numerous small, yellow lesions distributed throughout the posterior pole and midperiphery. Because FA is not routinely performed in patients with intermediate AMD and was not a part of the Amish Eye Study, this modality was not available in our cohort. Instead, cuticular drusen in this study were identified by examination of all B-scans in the OCT volume, and were deemed to be present when a lesion exhibited the characteristic morphologic patterns described in prior literature [9]: (1) shallow elevations of the RPE–basal lamina (BL) complex without clearly discernible internal material; (2) the classic saw-tooth configuration with a hyporeflective internal core; or (3) broad, mound-shaped RPE–BL elevations containing a homogenous medium-hyporeflective interior (Fig. 1). It is this relatively hyporeflective interior which creates the classic “donut-like” appearance when visualized on en face OCT (Fig. 1). Hyper-transmission tails on OCT, which have been recognized as a characteristic feature of cuticular drusen, were also required in our diagnostic criteria. Presence or absence of cuticular drusen was evaluated by 2 independent graders (JH and CC) on the basis of multimodal imaging (FAF, OCT, CFP), though the OCT features described above were the key features used for determination. Discordant diagnosis was resolved through open arbitration, with a third senior grader (S.S) making the final decision if the initial graders could not agree.

### Annotation of cuticular drusen on en face OCT

En face OCT images were obtained using the manufacturer’s multilayer segmentation software. A 20-μm-thick slab positioned from 30 to 50 μm above Bruch’s membrane was generated. Cuticular drusen could be recognized as hyperreflective ring-like (“donut”) lesions on this slab, though the hyporeflective core could be nearly pinpoint on small lesions (in which case the lesion appeared largely as a hyperreflective dot). A previous study [18] also showed that cuticular drusen manifested as donut sign on a 40 μm slab from 30–70 μm above Bruch’s membrane, though due to the hypertransmission tail, this became a reverse donut on the deeper choroidal en face slab. That previous study, however, used a different OCT device, and we empirically observed that our modified slab (as described above), best highlighted cuticular drusen, particularly for quantification. En face OCT images in this study were used as a spatial guide to facilitate the localization of individual drusen. Once the boundaries of the slab were chosen as noted above, each en face image was analysed for the distribution pattern of cuticular drusen in ImageJ.

Each candidate druse was verified by inspection of the corresponding cross-sectional OCT B-scan, and the coordinate positions of each druse was recorded. The position of the foveal centre was also marked and recorded using this same coordinate system. To ensure spatial comparability between eyes, coordinates from the left eyes were mirrored across the vertical axis so that nasal–temporal orientation was standardized across all eyes prior to analysis. The early treatment diabetic retinopathy study (ETDRS) grid was overlaid on the 6 × 6 mm en face OCT image. For the purpose of this analysis, only the concentric circles were used: a central circle of 1 mm diameter, an inner circle of 3 mm diameter, and an outer circle of 6 mm diameter. These circles divide the macula into 3 regions: a central foveal region, an inner ring (1–3 mm), and an outer ring (3-6 mm) [19]. The following parameters were measured in this analysis: 1) the relative location of each druse to the foveal centre which in aggregate for an eye, provides a measure of the topographical distribution of the cuticular drusen; 2) the relative distribution density of cuticular drusen within the 3 defined regions of the macular ETDRS grid. The relative distribution density was calculated by normalizing the number of manually confirmed cuticular drusen within each ETDRS region to the physical area of that region. Thus, for each eye, the total number of drusen within the central 1-mm circle, the inner 1–3-mm ring, and the outer 3–6-mm ring was divided by the corresponding area (0.79 mm², 6.28 mm², and 21.21 mm², respectively), yielding a density measure expressed as counts per mm².

### Grading of AMD

AMD staging was determined based on the Beckman classification system [14]. Intermediate AMD was defined as the presence of large drusen (≥125 μm) or the presence of pigmentary abnormalities in at least 1 eye. Late AMD is defined as (1) the presence of MNV, determined by the Consensus on Neovascular AMD Nomenclature criteria [20] or (2) the occurrence of geographic atrophy or cRORA as detected on CFP or OCT [21], or areas of decreased autofluorescence observed on fundus autofluorescence [22]. SDDs were characterized as mounds or cone-shaped structures with medium to high reflectivity, located either at the ellipsoid zone or between the ellipsoid zone and the RPE surface [23].

### Spatial feature extraction and clustering analysis

To characterize eye-level spatial distribution patterns of cuticular drusen, five features were derived from drusen coordinates relative to the fovea: mean radial distance, standard deviation of radial distance, proportion of drusen within the central ETDRS region, mean nearest-neighbour distance, and spatial variance. All features were standardized (z-scores) prior to clustering. K-means clustering was applied to identify groups with similar spatial patterns. Clustering was performed using Euclidean distance, which partitions observations by minimizing within-cluster sum of squares while maximizing between-cluster separation. The optimal number of clusters (*K* = 3) was determined using the elbow method based on within-cluster sum of squares (Supplementary Fig. 1), and clustering stability was ensured with multiple random initializations (n_start = 25). Clusters were interpreted as spatial phenotypes: centraldominant, outer-macular-dominant, and diffuse. Each eye was assigned to a single cluster, and the three spatial phenotypes were mutually exclusive by design. To assess whether the distributions were centred on the fovea, per-eye centroids were calculated as the mean x- and y-coordinates of all drusen, and group-level centroids were obtained by averaging across eyes within each cluster.

### Statistical analysis

Continuous variables are presented as mean ± standard deviation. Categorical variables are presented as counts and percentages. Group differences in categorical variables were assessed using Fisher’s exact test. Associations between clusters and AMD progression were evaluated using Firth’s penalized logistic regression, a bias-reduction approach suitable for rare binary outcomes that mitigates separation issues in conventional logistic models. Statistical analysis was performed using R software (R Foundation for Statistical Computing, Vienna, Austria). A *P* value of 0.05 or less was considered statistically significant.

## Results

88 eyes of 58 participants were included in this analysis. All participants except for 1 (1 eye) completed the 2-year follow up. The mean age of this cohort was 70.3 ± 9.6 years, and 34 patients were women (59.6%).

Quantitative analysis of drusen density across ETDRS regions demonstrated that cuticular drusen were most concentrated near the foveal centre, with density gradually decreasing toward the outer macula. The mean (SD) density of cuticular drusen was 6.14 (3.89) count/mm² in the central 1-mm region, 2.15 (1.18) count/mm² in the inner ring (1–3 mm), and 1.18 (0.81) count/mm² in the outer ring (3–6 mm).

**Fig. 2:**
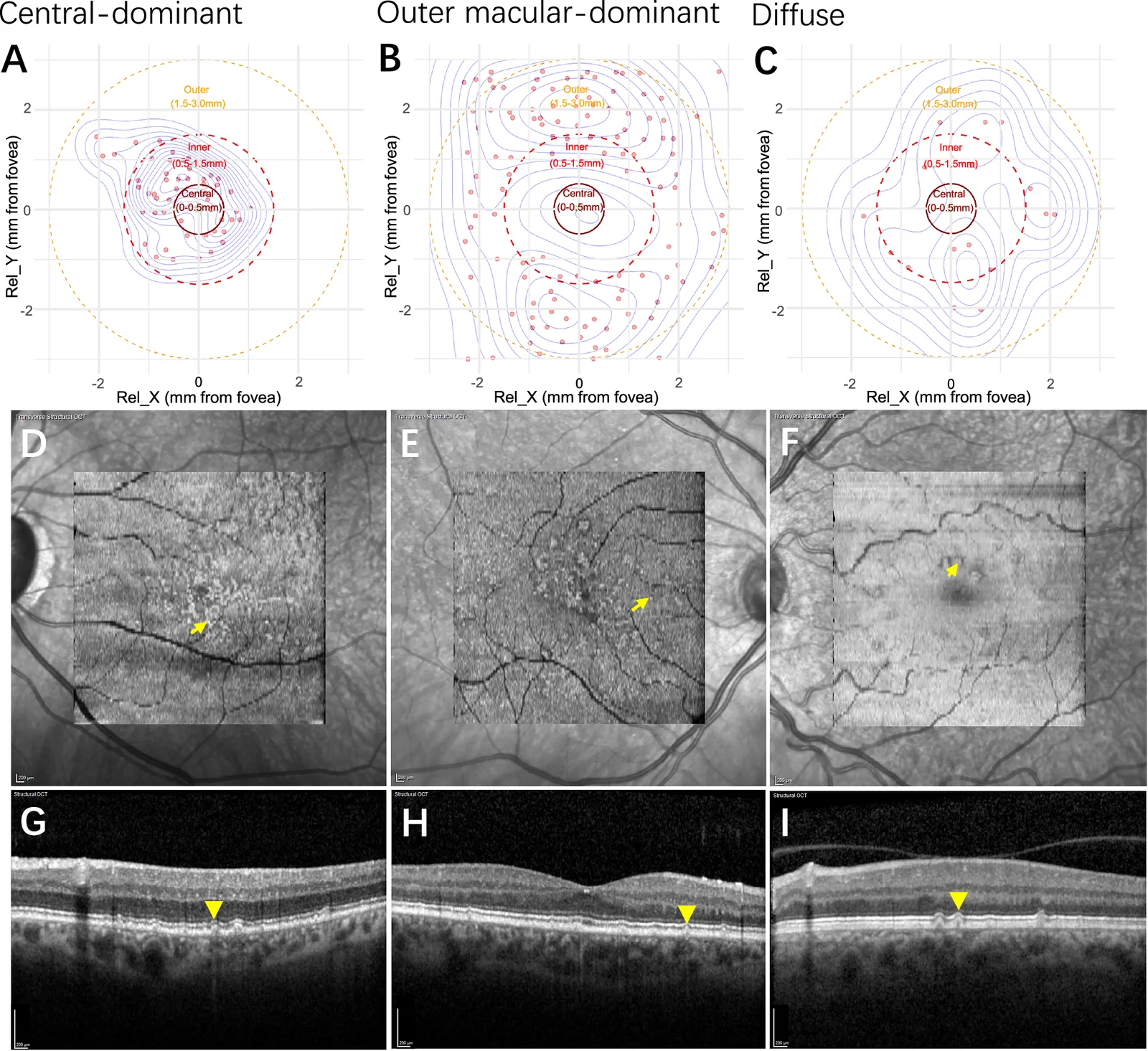
Representative figures of different phenotypes of cuticular drusen. **A**–**C** cluster plots of central-dominant, outer-macular-dominant and diffuse phenotype; **D**–**F** en face images of representative eyes from central-dominant, outer-macular-dominant and diffuse phenotypes; **G**–**I** OCT B scan of eyes from central-dominant, outer maculardominant and diffuse phenotypes. Arrows: cuticular drusen on en face OCT; Arrow heads: cuticular drusen on OCT B scans.

Unsupervised K-means clustering based on spatial distribution features identified three spatial phenotypes of cuticular drusen: central-dominant (50 eyes, 57.5%), outer maculardominant (28 eyes, 32.2%), diffuse (9 eyes, 10.3%). The central-dominant phenotype represented the largest proportion of eyes in the cohort. Representative multimodal images and schematic distribution patterns for each phenotype are shown in Fig. 2.

**Fig. 3:**
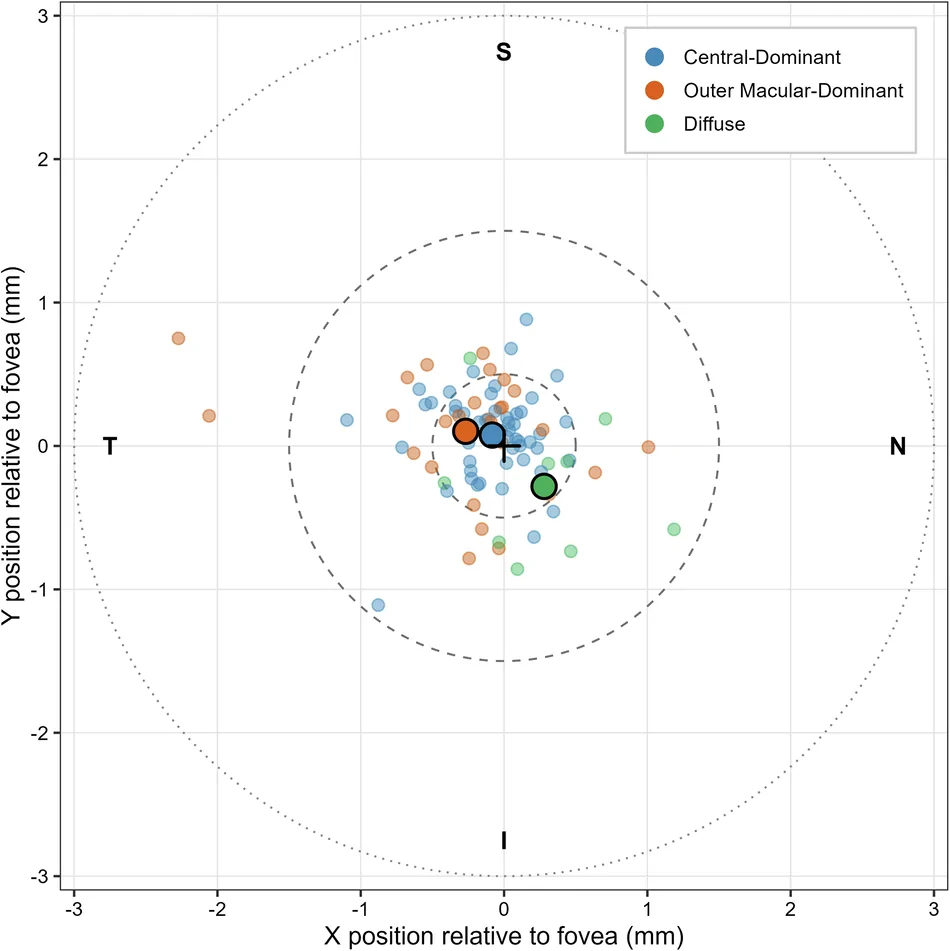
Centroid analysis of cuticular drusen spatial phenotypes. Each point represents the centroid (mean x–y location) of drusen distribution for an individual eye. Larger markers indicate the group-level mean centroid for each phenotype. The foveal centre is defined as (0,0). Left eyes were mirrored to match right-eye orientation.

Centroid analysis showed that all three spatial phenotypes had mean centroids located within the inner ring. The central-dominant phenotype exhibited a centroid closest to the foveal centre, whereas the outer-macular-dominant and diffuse phenotypes showed modest spatial offsets from the fovea (Fig. 3).

At baseline, no significant differences were observed in the prevalence of SDDs or large drusen across the three spatial phenotypes (Table 1).

**Table 1:** Baseline proportion of large drusen and SDDs in different phenotypes.

Cluster Name (Number)	Large drusen_BL (%)	SDDs_BL (%)
Central-dominant (50)	27 (54%)	7 (14.0%)
Outer macular-dominant (28)	19 (67.9%)	4 (14.3%)
Diffuse (9)	7 (77.8%)	3 (33.3%)
*P* value	0.32	0.35

*BL* baseline, *SDDs* subretinal drusenoid deposits.

During the 2-year follow-up, 5 eyes progressed to late AMD, including 4 eyes in the outer-macular-dominant group and 1 eye in the central-dominant group, while no progression events occurred in the diffuse group. To further explore this observation, the outer-macular-dominant group was defined as a high-risk group, while the central-dominant and diffuse groups were combined as a low-risk group. In unadjusted Firth logistic regression, the high-risk group was associated with increased odds of progression (OR 7.16, 95% CI 1.24–74.20, *p* = 0.027). After adjustment for SDDs and large drusen, this association remained directionally consistent (OR 11.53, 95% CI 1.62–169.00, *p* = 0.013).

In the adjusted model, SDDs also showed an association with progression (OR 9.26, 95%

CI 1.36–78.65, *p* = 0.023), whereas large drusen was not associated with progression (*p* = 0.491) (Table 2). These results should be interpreted cautiously given the low number of progression events and the wide confidence intervals, particularly in the adjusted model.

**Table 2:** Firth logistic regression analysis for 2-year progression to late AMD.

Model	Variable	OR	95% CI	*P* value
**Unadjusted**	High-risk group vs low-risk group	7.16	1.24–74.20	0.027*
**Adjusted**	High-risk group vs low-risk group	11.53	1.62–169.00	0.013*
**Adjusted**	SDDs	9.26	1.36–78.65	0.023*
**Adjusted**	Large drusen	0.5	0.06–3.94	0.49

*OR* odds ratio.

High-risk group: the outer macular-dominant phenotype.

Low-risk group: the central-dominant and diffuse phenotypes.

*: statistically significant.

A sensitivity analysis restricting the dataset to one eye per participant showed a similar direction of association (OR 16.33; 95% CI 1.46–2243.98; *p* = 0.021).

## Discussion

In this study, quantitative analysis of en face OCT images identified three spatial phenotypes of cuticular drusen within the macula: central-dominant, outer maculardominant, and diffuse. Eyes exhibiting an outer-macular-dominant phenotype demonstrated a higher rate of progression to late AMD over two years compared with other phenotypes; however, this association should be interpreted as exploratory given the small number of progression events. To our knowledge, this study was the first to apply en face OCT to describe the distribution of cuticular drusen and its relationship of progression of AMD. The differential risk for progression based on the distribution may help explain inconsistencies with regards to progression risk in prior studies.

Previous studies [4, 9, 24] have characterized cuticular drusen using multimodal imaging. On fundoscopy, they appear as small, round, yellowish sub-RPE deposits [25], while fluorescein angiography demonstrates the characteristic “stars-in-the-sky” pattern [2]. On OCT, cuticular drusen typically present as small RPE elevations, often with a “sawtooth” configuration [2, 26], although this feature is not universally present [27]. Additional OCT features include shallow or mound-shaped RPE elevations [9] and, in some cases, hypertransmission tails or barcoding appearance extending into the choroid [10, 27, 28]. In the present study, fluorescein angiography was not available in the AMISH Eye Study cohort; therefore, cuticular drusen were identified based on characteristic OCT features. To ensure high diagnostic certainty for spatial analysis, we restricted inclusion to lesions demonstrating hypertransmission tails. While this approach improves diagnostic certainty, it may preferentially select a subset of lesions with distinct structural or biological characteristics. As a result, the spatial patterns observed in this study may not fully represent the distribution of all cuticular drusen. In addition, if such lesions are associated with disease progression, this selection may influence the observed relationship between spatial phenotype and progression risk. Future studies including a broader spectrum of lesion morphology are warranted to determine the generalizability of these findings.

Topographic distribution of cuticular drusen using qualitative classification have been described by different studies. Balaratnasingam et al. [9] categorized distributions as macular (32.9%) or diffuse (67.1%), whilst Shin et al. [29] reported a similar predominance of the diffuse pattern (61.7%). Sakurada et al. [4] further defined three phenotypes (concentrated, scattered, and mixed) and Paolo et al. [30] demonstrated that cuticular drusen were most frequently located within the inner ETDRS ring. In contrast, we used en face OCT to perform a quantitative assessment of drusen distribution by annotating the number and location of individual lesions. Though it was a laborious process, we labelled the number and position of each druse on the en face image, to allow for a more objective description of the distribution within the macula as en face OCT may offer practical advantages, as it allows rapid visualization of drusen distribution across the macula.

The role of cuticular drusen as a risk factor for progression to late AMD remains controversial. Several studies [10, 31] have reported no independent association with progression, whereas others have suggested an increased risk [32], particularly in the presence of coexisting features such as SDDs and large drusen [4, 33]. A recent analysis by our team [11] found that at least through 2 years, cuticular drusen did not independently increase the risk for developing late AMD. Our study showed that eyes with an outer-macular-dominant distribution of cuticular drusen showed a higher rate of progression to late AMD over two years compared with central-dominant and diffuse phenotypes, which is broadly consistent with the results reported by Sakurada et al. [4], who demonstrated that phenotype 2 (scattered distribution) was associated with a higher 5-year risk of progression to late AMD compared with phenotype 1 (concentrated distribution). However, the present study is retrospective with a relatively short 2-year follow-up, which may limit the strength of temporal inference, particularly in comparison with the prospective design and longer follow-up in Sakurada et al. After adjustment for established risk factors, SDDs remained associated with progression, whereas large drusen were not associated with progression in this cohort. It should be noted that large drusen were defined using a threshold of >125 μm in our study, compared with >200 μm in Sakurada et al. These findings suggest that spatial distribution patterns of cuticular drusen may capture aspects of disease risk not fully reflected by conventional structural biomarkers. A more peripheral distribution reflects a shift in lesion location rather than greater areal involvement; thus, the increased risk observed in the outer macular-dominant phenotype may not be explained by lesion extent alone. An alternative explanation is that peripheral localization reflects region-specific vulnerability of the RPE or choroidal circulation. Spatial variation in metabolic demand or perfusion across the macula may contribute to differential susceptibility to progression, suggesting that lesion distribution may serve as a marker of underlying biological heterogeneity rather than disease burden alone. In addition, centroid analysis demonstrated that these spatial phenotypes were not strictly centred on the fovea, but exhibited modest spatial offsets, indicating that cuticular drusen distribution is not purely concentric. A similar spatial bias has been described in SDDs, which preferentially localize to the superior macula and have been hypothesized to relate to choroidal perfusion gradients and gravitational effects [34]. However, whether similar mechanisms underlie cuticular drusen distribution remains unclear. Further studies are needed to elucidate the biological basis and clinical implications of these spatial patterns.

Limitations of our study include the retrospective design and a relatively small sample size. In addition, only five progression events occurred in the cohort, and the corresponding confidence intervals were wide, particularly in the adjusted model, which limited the precision of the effect estimates. Therefore, the observed association between the outer-macular-dominant phenotype and progression to late AMD should be considered exploratory. To validate our conclusions regarding the risk of late AMD, future investigations will necessitate a longer follow-up period with larger cohorts. Furthermore, the manual labelling of cuticular drusen introduces the potential for subjective bias. The future implementation of automated analysis algorithms will be essential for mitigating this potential source of error and concurrently reducing the required workload. Our methodology involved setting the specific thickness and boundaries of the en face slab based on a subjective assessment to achieve optimal visualization of cuticular drusen. Additional studies using different segmentation slabs will be important to further validate our findings. Finally, the current study was limited to analysing the distribution of cuticular drusen within the macular area due to the use of a standard 6*6 mm OCT scanning range.

Subsequent research utilizing wide-field OCT is warranted to analyse distribution patterns across a broader area of the retina, and to determine who these broader patterns might influence progression risk. Despite these limitations, this study demonstrates the feasibility of quantitatively characterizing spatial phenotypes of cuticular drusen using en face OCT. Identification of distinct spatial patterns may help explain heterogeneity in reported progression risk among patients with cuticular drusen and may contribute to improved phenotypic stratification in future AMD studies.

## Conclusions

Quantitative analysis of en face OCT images demonstrated that cuticular drusen are most densely distributed near the foveal centre within the macula. Using unsupervised clustering of spatial features, three spatial phenotypes were identified—central-dominant, outer-macular-dominant, and diffuse—with the central-dominant pattern being the most prevalent.

Eyes with an outer-macular-dominant phenotype showed a higher rate of progression to late AMD over two years than the other two phenotypes. In unadjusted analyses, this phenotype was associated with higher odds of progression, after adjustment for SDDs and large drusen, the association between spatial phenotype and progression remained.

Overall, an outer-macular-dominant cuticular drusen distribution may be associated with an increased risk of 2-year progression to late AMD in this exploratory analysis. Together with established biomarkers such as large drusen and SDDs, this phenotype may warrant further evaluation as a potential independent risk marker for advanced AMD.

## Summary

### What was known before

- Cuticular drusen are a distinct AMD phenotype, but their impact on the risk of disease progression remains controversial.
- Previous classifications of cuticular drusen have been largely qualitative, lacking precise quantitative analysis of their spatial distribution.

### What this study adds

- This study uses en face OCT and cluster analysis to identify three distinct spatial patterns of cuticular drusen: central-dominant, outer-macular-dominant, and diffuse.
- Eyes with an outer-macular-dominant phenotype showed a higher rate of progression to late AMD than other phenotypes, suggesting that this distribution pattern may warrant further evaluation as a potential quantitative risk marker for advanced AMD.

## Supplementary information

## Data Availability

The datasets generated during and/or analysed during the current study are available from the corresponding author on reasonable request.
