# Supplementary material for: Spatial distribution of cuticular drusen and its association with category-specific progression risk in intermediate AMD
Source: Eye (Lond). 2026 Jun 19;40(13):2054–60. doi: 10.1038/s41433-026-04631-w (PMC13533437; doi:10.1038/s41433-026-04631-w)
Supplement: Supplementary file 1 [file 41433_2026_4631_MOESM1_ESM.pdf]

Supplementary figure legend

Supplementary figure 1. Elbow method plot showing the within-cluster sum of squares (WCSS) across different numbers of clusters (K), with K = 3 selected as the optimal clustering solution.
